# Getting the Measure of Positional Information

**DOI:** 10.1371/journal.pbio.1000081

**Published:** 2009-03-31

**Authors:** Johannes Jaeger, Alfonso Martinez-Arias

## Abstract

Quantitative measurements and mathematical modeling finally allow us to probe the limits of precision in developmental systems and reveal the importance of feedback regulation for developmental robustness.

## Of Fruit Flies and French Flags

**Figure 1:**
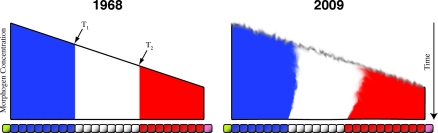
The French Flag Model Now and Then Left: Wolpert's original. Thresholds T_1_ and T_2_ in the concentration of a morphogen gradient determine the territories of target gene expression (indicated by blue, white, and red). Each threshold corresponds exactly to an expression boundary. Right: Revised version. Expression boundaries move after their initial establishment by the gradient (indicated by time axis on the right), and low precision in the gradient and the initial target gene boundaries (illustrated by fuzzy borders) become sharpened due to downstream regulatory interactions.

Understanding the mechanisms that underlie pattern formation is one of the major challenges of developmental biology. The complexity and beauty of the patterns on butterfly wings, fish scales, or bird feathers are not only remarkable products of developmental processes but puzzles that tease our intellects. If we are to understand these beautiful products of cellular activity, we need to first investigate simpler patterns, which are more tractable experimentally. A good example is the subdivision of an embryo along its main axis, which can be represented as a polarized subdivision of a cellular field. Over 40 years ago, Lewis Wolpert offered a conceptual solution to this problem in the form of the French Flag model [1]. The central element of the proposal is that spatial gradients of substances called morphogens are the cause of such subdivision (Figure 1, left panel). The idea is simple: specific concentration thresholds in the gradient are detected by cells in the target tissue and lead to the expression of distinct sets of target genes. The crucial ingredient of the argument was a precise and direct correlation between the input (the gradient) and the output (the response of the tissue)—each threshold corresponds precisely to a border of an expression territory.

**Figure 2:**
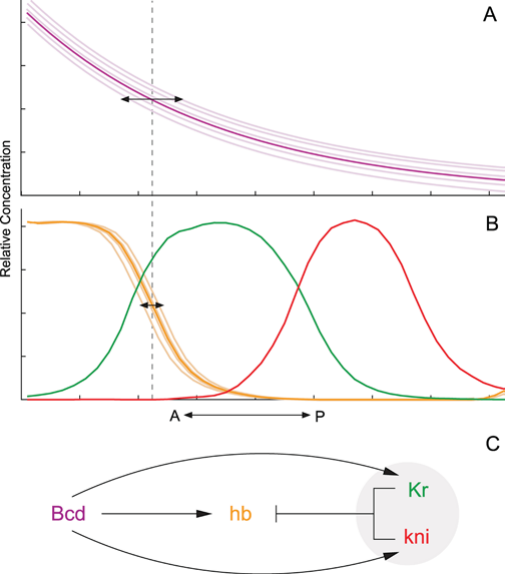
Quantified Expression Data and Mathematical Modelling Reveal Additional Interactions Required for hb Precision Spatial profiles of (A) Bcd (magenta) as well as (B) Hb (orange), Kr (green), and Kni (red) are shown. Light-coloured profiles and double-headed arrows indicate embryo-to-embryo variability in Bcd and Hb. (C) Network diagram representing regulatory interactions required for *hb* precision. The gray circle highlights additional gap repressors.

However, the quantitative nature of the argument made it difficult to test for the lack of adequate measurable observables. The discovery of the Bicoid (Bcd) gradient in the early embryo of the fruit fly Drosophila melanogaster in the 1980s (Figure 2A) provided the first direct evidence for the existence of the postulated morphogen gradients [2–4]. Bcd is a transcription factor that—within the syncytium of the early Drosophila embryo—forms a concentration gradient from anterior to posterior. The gradient is necessary for the antero-posterior patterning of the embryo [3]. This finding led to a general revival of interest in spatial patterning and to the discovery of many similar gradients involved in other developmental processes, such as patterning of the wing disc in Drosophila or the neural tube in vertebrates [5,6].

Bcd activates transcription of gap genes such as *hunchback* (*hb*) (Figure 2B) in a concentration-dependent manner [7,8]. This observation—together with the more general correlation between the concentration of Bcd and downstream expression patterns [3]—provided support for the hypothesis that Bcd is a morphogen [9,10]. If this were the case, Bcd alone should be capable of precisely positioning boundaries of downstream gene expression as proposed by the French Flag. But is it? To test this requires precise measurements of Bcd and its targets.

One major issue, first highlighted by C. H. Waddington, is the heavy reliance of the French Flag model on precise detection of concentration thresholds by cells in the target tissue [9]. Early efforts to model the system indicated that target gene auto-activation [11], or more complex interactions among downstream factors [12,13], would be necessary to achieve the required robustness. However, these studies were hampered by the absence of reliable experimental evidence on the variability of gene expression against which the models could be tested.

## Quantification at Last!

Developmental biology is changing. Qualitative, descriptive approaches are beginning to yield to quantitative, analytical ones, through the development of optical and analytical techniques. These approaches allow us to state more precise hypotheses (formulated as predictive models), which can be tested more rigorously. Many of these techniques have been applied to Bcd. In a first set of observations, Houchmandzadeh et al. [14] measured the variability in the spatial expression of Bcd and Hb across a large number of individual embryos. This was achieved by collecting quantitative expression profiles for both Bcd and Hb proteins and measuring the spatial error caused by fluctuations in protein levels between individual embryos. This error was shown to be large for Bcd, whereas it was very small for *hb* at the position of its posterior boundary (Figure 2A and 2B, dashed line). This is not what the French Flag model predicts! The apparent increase in positional precision is independent of mutations in other segmentation genes, with the notable exception of certain alleles of *staufen* (*stau*), a gene that is involved in localisation of maternal mRNAs. These results indicated that regulation by Bcd is not sufficient for *hb* precision, and led to the theoretical prediction of a missing posterior regulator gradient [15–17], or active *hb* mRNA transport involving the Stau protein [18]. At the moment, there is no experimental evidence supporting either of these proposed mechanisms.

A subsequent study by Crauk and Dostatni [19] contradicted the above results by showing that a reporter construct, which only contains Bcd binding sites, and even reporters responding to heterologous gradients of yeast GAL4 transcription factor, are expressed with high levels of precision. More-recent measurements of the Bcd gradient in vivo (using a Bcd-GFP fusion) or using immunofluorescence reveal that the positional error quantified by Houchmandzadeh et al. [14] was exaggerated by the way expression data were normalised in the original paper [20–22]. In addition, one of these studies shows that Bcd variability is greatly increased in *stau* mutants [22]. This suggests that Bcd, while variable, is more precise than previously thought and that this precision is necessary for accurate positioning of the *hb* border. But, is it sufficient as well?

An elegant theoretical analysis—using Berg and Purcell's theory of the precision of gradient sensing in bacteria [23]—indicated that for the observed precision to be achieved, target cells need to measure Bcd concentration for several hours [21]. This is clearly not possible, as the Hb expression pattern is established within about 20 minutes. Furthermore, the corrected measurements indicate that the observed error in Bcd concentration at the relevant threshold is still about twice as high as that of Hb [22,24] (Figure 2A and 2B). Different hypothetical mechanisms—based on spatial averaging of regulatory input from Bcd to *hb* [21] or correlations between scaling of the Bcd gradient and sensitivity of *hb* to Bcd [22]—have been proposed to account for the remaining discrepancy, but the proposed solutions are not satisfactory, because their mechanistic basis remains elusive.

## Precision Due to Downstream Interactions

Two new papers by Manu et al., one in *PLoS Biology* (doi:10.1371/journal.pbio.1000049) and the other in *PLoS Computational Biology* (doi:10.1371/journal.pcbi.1000303) [25,26], now shed new light on the issue of Bcd versus Hb precision. Bcd is not the only regulatory input into *hb*, which is repressed by the gap genes *Krüppel* (*Kr*) and *knirps* (*kni*) [27,28] (Figure 2B and 2C). While earlier studies had only tested the effect of single gap gene mutants on the expression of *hb* [14], or completely ignored gap–gap cross-regulation [19–22], Manu et al. [25] show that *Kr kni* double mutants show increased variability in the position of the *hb* domain boundary, which suggests that their interactions with *hb* are required for precise positioning of its boundary.

But how can gap–gap cross-regulation increase spatial precision? Detailed mathematical models of the entire gap gene system—represented as an integrated gene circuit—are required to address this question. Manu et al. [25] obtained such models by fitting them to quantitative expression data. Analysis of the resulting gene circuits reveals that the reduced error in boundary placement is due to feedback in regulatory interactions: Bcd not only activates *hb* but also its repressors *Kr* and *kni* (Figure 2C). If there is more Bcd in an embryo, there is also more repressor effectively cancelling the increased maternal activation. In *Kr kni* double mutants, this compensation is missing, and *hb* varies precisely as expected if it were to depend on Bcd alone. This begins to shed some light from a familiar and measured perspective.

A second paper by the same authors [26] uses a more rigorous mathematical approach to show how this regulation comes about and, in the process, illustrates how the methodology of complex systems theory [29] can be used to investigate the dynamics of real developmental gene regulatory networks. The authors analyse the dynamical structure, or state space, of their model. State space is an imaginary volume with (in this case) regulator concentrations as its dimensions. Dynamical systems are governed by stable points (called attractors) in their state space towards which they will converge, as long as their initial conditions lie within a given set of concentrations (a basin of attraction). In this way, the structure of its basins of attraction will determine the dynamical behaviour of the system. Abstract as this may sound to a biologist, it is a good way to represent a complex system in which multiple variables interact over time to produce an output.

Specifically, the structure of state space can be used to explain expression features, such as gap domain boundaries. There are different ways in which such a boundary can form [26]. In the case of *hb*, it is formed by the system falling into two different basins of attraction on each side of the boundary. Nuclei lying anterior will tend towards an attractor expressing *hb*, while nuclei lying more posterior will express *Kr* instead. In contrast, more posterior nuclei never reach their respective attractors, but instead converge onto a common trajectory—called an unstable transient manifold—which explains why posterior gap domains shift their position towards the anterior of the embryo over time [30].

Manu et al. [26] show that precision of the system can be explained by an interesting feature of its attractors and its transient manifold: they contract the state space the system can occupy. Although the system may start at different initial conditions in different embryos (because of variability in the Bcd gradient), it will converge very rapidly to similar combinations of regulator concentrations and thus to very restricted sub-regions of state space. This compaction explains how precision can increase in any feedback-driven developmental system and confirms—in a rigorous, mathematical way—the observation made by C. H. Waddington in 1942 that developmental trajectories are buffered against perturbations, a phenomenon he called canalization [31].

In contrast to earlier studies, the Manu et al. [25,26] papers account for the precision of many gap domain boundaries (not just *hb*) and provide a consistent interpretation of the various, seemingly contradictory, experimental results mentioned above. They account for the residual discrepancy between Bcd and Hb precision [21,22]. They also explain the results of Crauk and Dostatni [19], since the model predicts that precision increases towards the anterior end of the embryo, where the boundaries of the reporter constructs used in that study occur. In our view, these manuscripts provide a satisfactory answer to some of the puzzles that have been lingering for a number of years; however, several important questions remain. For instance, while the mechanism presented can reduce embryo-to-embryo variability in monotonically decreasing concentrations of Bcd, it seems unable to reduce non-monotonic stochastic fluctuations between nuclei in individual embryos. It is likely that quantitative modelling studies using stochastic formalisms will be required to address such issues.

## Robustness through Feedback

Why are the insights gained by these studies important and exciting? Quantitative measurements allow us to test models in detail, particularly when real data are available, and to reveal their limitations. In the case of the French Flag, models show that regulatory interactions among target genes are essential for both positional specification and the robustness of pattern formation. These interactions cause gap domain boundaries to shift and are required to convert noisy early patterns into precise late ones (illustrated in Figure 1, right panel) [25,26,30]. We suspect that such feedback-driven mechanisms had not been considered before, because it is impossible to study them using the traditional, qualitative methods of genetics and molecular biology. They can only be investigated using quantitative measurements and mathematical modelling.

Furthermore, the insights gained from the early Drosophila embryo are widely applicable to other systems. Similar approaches have shown that gradient precision in the wing disc is equally insufficient to account for the accurate positioning of target domain boundaries [32], and at least three levels of feedback are involved in setting target domain boundaries in the developing vertebrate neural tube [6,33]. These examples indicate that complex feedback is likely to be very common in development, and that it is required to account for the observed robustness of developmental systems [34].

The studies discussed above acknowledge that measurements can provide insights into the mechanisms that determine patterns during development. They begin to shift our focus from the study of average or typical patterns to appreciate the implications of the intrinsic variability of pattern formation in individuals and, in particular, the requirements it imposes on control mechanisms. While the example above illustrates a system in which fluctuations have to be restricted, there is emerging evidence that noise may play a more constructive role in development as well [35]. This opens new and exciting possibilities for developmental biologists, and fills us with the hope that we are witnessing only the beginning of a new era in the study of pattern formation.

## Abbreviations

Bcd: Bicoid
Hb: Hunchback
Kni: Knirps
Kr: Krüppel
Stau: Staufen

## References

[pbio-1000081-b001] Wolpert L, Waddington CH (1968). The French Flag problem: A contribution to the discussion on pattern development and regulation. Towards a theoretical biology,.

[pbio-1000081-b002] Driever W, Nüsslein-Volhard C (1988). A gradient of *bicoid* protein in Drosophila embryos. Cell.

[pbio-1000081-b003] Driever W, Nüsslein-Volhard C (1988). The *bicoid* protein determines position in the Drosophila embryo in a concentration-dependent manner. Cell.

[pbio-1000081-b004] Ephrussi A, St Johnston D (2004). Seeing is believing: the *bicoid* morphogen gradient matures. Cell.

[pbio-1000081-b005] Tabata T, Takei Y (2004). Morphogens, their identification and regulation. Development.

[pbio-1000081-b006] Dessaud E, McMahon AP, Briscoe J (2008). Pattern formation in the vertebrate neural tube: a sonic hedgehog morphogen-regulated transcriptional network. Development.

[pbio-1000081-b007] Struhl G, Struhl K, Macdonald PM (1989). The gradient morphogen *bicoid* is a concentration-dependent transcriptional activator. Cell.

[pbio-1000081-b008] Driever W, Nüsslein-Volhard C (1989). The *bicoid* protein is a positive regulator of *hunchback* transcription in the early Drosophila embryo. Nature.

[pbio-1000081-b009] Wolpert L (1989). Positional information revisited. Development.

[pbio-1000081-b010] Wolpert L (1996). One hundred years of positional information. Trends Genet.

[pbio-1000081-b011] Lewis J, Slack JMW, Wolpert L (1977). Thresholds in development. J Theor Biol.

[pbio-1000081-b012] Meinhardt H (1977). A model of pattern formation in insect embryogenesis. J Cell Sci.

[pbio-1000081-b013] Meinhardt H (1978). Space-dependent cell determination under the control of a morphogen gradient. J Theor Biol.

[pbio-1000081-b014] Houchmandzadeh B, Wieschaus E, Leibler S (2002). Establishment of developmental precision and proportions in the early Drosophila embryo. Nature.

[pbio-1000081-b015] Howard M, ten Wolde PR (2005). Finding the center reliably: Robust patterns of developmental gene expression. Phys Rev Lett.

[pbio-1000081-b016] Houchmandzadeh B, Wieschaus E, Leibler S (2005). Precise domain specification in the developing Drosophila embryo. Phys Rev E.

[pbio-1000081-b017] McHale P, Rappel W-J, Levine H (2006). Embryonic pattern scaling achieved by oppositely directed morphogen gradients. Phys Biol.

[pbio-1000081-b018] Aegerter-Wilmsen T, Aegerter CM, Bisseling T (2005). Model for the robust establishment of precise proportions in the early Drosphila embryo. J Theor Biol.

[pbio-1000081-b019] Crauk O, Dostatni N (2005). Bicoid determines sharp and precise target gene expression in the Drosophila embryo. Curr Biol.

[pbio-1000081-b020] Gregor T, Wieschaus EF, McGregor AP, Bialek W, Tank DW (2007). Stability and nuclear dynamics of the *bicoid* morphogen gradient. Cell.

[pbio-1000081-b021] Gregor T, Tank DW, Wieschaus EF, Bialek W (2007). Probing the limits to positional information. Cell.

[pbio-1000081-b022] He F, Wen Y, Deng J, Lin X, Lu LJ (2008). Probing intrinsic properties of a robust morphogen gradient in Drosophila. Dev Cell.

[pbio-1000081-b023] Berg HC, Purcell EM (1977). Physics of chemoreception. Biophys J.

[pbio-1000081-b024] Reinitz J (2007). A ten per cent solution. Nature.

[pbio-1000081-b025] Manu, Surkova S, Spirov AV, Gursky V, Janssens H (2009). Canalization of gene expression in the Drosophila blastoderm by gap gene cross regulation. PLoS Biol.

[pbio-1000081-b026] Manu, Surkova S, Spirov AV, Gursky V, Janssens H (2009). Canalization of gene expression and domain shifts in the Drosophila blastoderm by dynamical attractors. PLoS Comp Biol.

[pbio-1000081-b027] Jäckle H, Tautz D, Schuh R, Seifert E, Lehmann R (1986). Cross-regulatory interactions among the gap genes of Drosophila. Nature.

[pbio-1000081-b028] Clyde DE, Corado MSG, Wu X, Paré A, Papatsenko D (2003). A self-organizing system of repressor gradients establishes segmental complexity in Drosophila. Nature.

[pbio-1000081-b029] Strogatz S (2000). Nonlinear dynamics and chaos.

[pbio-1000081-b030] Jaeger J, Surkova S, Blagov M, Janssens H, Kosman D (2004). Dynamic control of positional information in the early Drosophila embryo. Nature.

[pbio-1000081-b031] Waddington CH (1942). Canalization of development and the inheritance of acquired characters. Nature.

[pbio-1000081-b032] Bollenbach T, Pantazis P, Kicheva A, Bökel C, González-Gaitán M (2008). Precision of the Dpp gradient. Development.

[pbio-1000081-b033] Dessaud E, Yang LL, Hill K, Cox B, Ulloa F (2007). Interpretation of the *sonic hedgehog* morphogen gradient by a temporal adaptation mechanism. Nature.

[pbio-1000081-b034] Jaeger J, Irons D, Monk N (2008). Regulative feedback in pattern formation: Towards a general relativistic theory of positional information. Development.

[pbio-1000081-b035] Martinez Arias A, Hayward P (2006). Filtering transcriptional noise during development: concepts and mechanisms. Nat Rev Genet.

